# Factors associated with improved outcome of inhaled corticosteroid use in COVID-19: A single institutional study

**DOI:** 10.1097/MD.0000000000032420

**Published:** 2022-12-23

**Authors:** Andrew Manfra, Claire Chen, Kavita Batra, Kyaw Min Tun, Mutsumi John Kioka

**Affiliations:** a Division of Pulmonary and Critical Care Medicine, Department of Internal Medicine, University of Nevada Las Vegas, Nevada, Las Vegas, USA; b Department of Medical Education and Office of Research, University of Nevada Las Vegas, Nevada, Las Vegas, USA.

**Keywords:** SARS-CoV-2, ARDS, inhaled corticosteroid, COVID-19, viral pneumonia

## Abstract

Asthmatics seem less prone to adverse outcomes in coronavirus disease 2019 (COVID-19) and some data shows that inhaled corticosteroids (ICS) are protective. We gathered data on anecdotal ICS and outcomes of patients hospitalized with COVID-19, given there is literature supporting ICS may reduce risk of severe infection. In addition, we fill gaps in current literature evaluating Charlson Comorbidity Index (CCI) as a risk assessment tool for COVID-19. This was a single-center, retrospective study designed and conducted to identify factors associated intubation and inpatient mortality. A multivariate logistic regression model was fit to generate adjusted odds ratios (OR). Intubation was associated with male gender (OR, 2.815; 95% confidence interval [CI], 1.348–5.881; *P* = .006) and increasing body mass index (BMI) (OR, 1.053; 95% CI, 1.009–1.099; *P* = .019). Asthma was associated with lower odds for intubation (OR, 0.283; 95% CI, 0.108–0.74; *P* = .01). 80% of patients taking pre-hospital ICS were not intubated (n = 8). In-patient mortality was associated with male gender (OR, 2.44; 95% CI, 1.167–5.1; *P* = .018), older age (OR, 1.096; 95% CI, 1.052–1.142; *P* = <.001), and increasing BMI (OR, 1.079; 95% CI, 1.033–1.127; *P* = .001). Asthma was associated with lower in-patient mortality (OR, 0.221; 95% CI, 0.057–0.854; *P* = .029). CCI did not correlate with intubation (OR, 1.262; 95% CI, 0.923–1.724; *P* = .145) or inpatient mortality (OR, 0.896; 95% CI, 0.665–1.206; *P* = .468). Asthmatics hospitalized for COVID-19 had less adverse outcomes, and most patients taking pre-hospital ICS were not intubated. CCI score was not associated with intubation or inpatient mortality.

## 1. Introduction

COVID-19 (Coronavirus Disease 2019), caused by the novel *SARS-CoV-2* virus (Severe Acute Respiratory Syndrome Coronavirus 2), was initially discovered in Wuhan, China, in December 2019.^[1]^ Surprisingly the characteristics of patients hospitalized during the onset of the COVID-19 pandemic in China did not reflect as many patients with asthma as expected. The prevalence of asthma in Wuhan was 6.4%, and only 0.9% of COVID-19 patients were discovered to have asthma.^[1]^ Viral respiratory infections have been notorious for triggering asthma exacerbations,^[2]^ including prior coronaviruses.^[3]^ However, observational studies have revealed no association between COVID-19 infection and increased risk for asthma exacerbations, hospitalization, or overall mortality.^[4–6]^ Since many patients with asthma use inhaled corticosteroids (ICS), there have been hypotheses that ICS are protective against COVID-19.^[7,8]^ Asthmatics taking anecdotal ICS have reduced rates of hospitalizations in COVID-19.^[9]^ A review of COVID-19 patients receiving ICS for asthma or chronic obstructive pulmonary disease before hospitalization had decreased rates of intubation.^[10]^ In our retrospective analysis, we gathered data on home ICS use to further describe the association between anecdotal ICS and COVID-19 outcomes.

Furthermore, studies evaluating at-risk groups for COVID-19 have noted numerous comorbid conditions associated with adverse outcomes.^[11–14]^ Patients with multiple comorbidities have significantly increased hospitalization rates and mortality compared to patients with few or no comorbid conditions.^[12,13]^ The Charlson Comorbidity Index (CCI) was created as a validated method for estimating the risk of death from comorbid diseases for use in longitudinal studies.^[15]^ CCI considers the number and seriousness of comorbid conditions, with each increase in CCI corresponding to a step-wise increase in overall mortality from comorbid disease. Given that an increasing number of comorbid conditions are associated with increased mortality in COVID-19, we assessed if CCI is effective in predicting the risk of intubation or in-patient mortality in the setting of COVID-19 illness. No studies have evaluated whether this validated risk assessment tool is effective in COVID-19.

The current study aims to describe the characteristics, outcomes, and hospital resource utilization of COVID-19 patients during the early months of the pandemic in Las Vegas, Nevada. Our study fills gaps in current literature evaluating CCI as a risk assessment tool for COVID-19 illness. In addition, we provide further data evaluating the correlation between home ICS use and outcomes of patients hospitalized with COVID-19 illness, given the literature supporting ICS use may be protective against COVID-19 adverse outcomes.

## 2. Methods

### 2.1. Study design and setting

This study was a retrospective cohort and single institutional study. Patients’ medical charts were reviewed, and pertinent data for this study were collected from March 1, 2020 to November 30, 2020, at an urban-based tertiary care teaching hospital in Nevada. This study was approved with a waiver of informed consent by the Institutional Review Boards of the University of Nevada Las Vegas (IRB No 1723786-1) and University Medical Center of Southern Nevada, Las Vegas, Nevada (IRB No UMC-2021-335).

### 2.2. Selection criteria of the sample and measures

**Figure 1.**
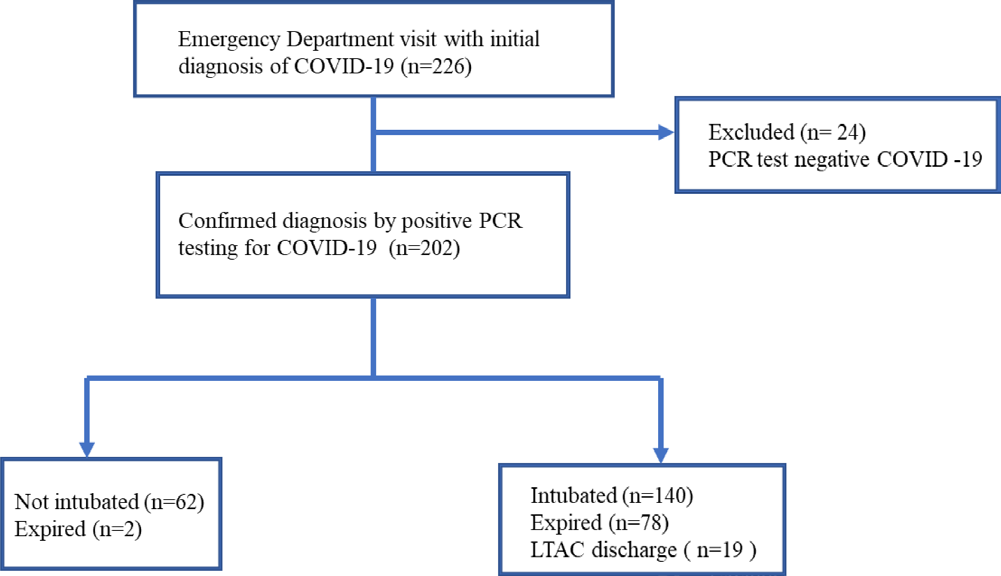
Cohort study flow diagram. COVID-19 = coronavirus disease 2019, LTAC = long term acute care, PCR = polymerase chain reaction.

Patients aged ≥ 18 years and those with a definitive diagnosis of COVID-19 infection were included. The clinical diagnosis was made by positive COVID-19 polymerase chain reaction (PCR) testing from a nasal or oropharyngeal swab obtained in the emergency department. Subjects with negative COVID-19 PCR testing and pregnant patients were excluded from the study. A detailed sample selection process is shown in Figure 1. Patients were categorized into 2 groups, intubated or not intubated. Comorbidities and home ICS were identified by manual chart review of historical documentation on admission or the patient’s hospital problem list. CCI^[15]^ and systemic inflammatory response syndrome criteria^[16]^ were obtained from the initial presentation to the emergency department as documented in the history and physical exam. CCI was further classified into 5 categories correlating with predicted 10-year survival. The categories were as follows: Category 0 = 0 points; 98% 10-year survival, Category 1 = 1 to 2 points; 90% to 96% 10-year survival, Category 2 = 3 to 4 points; 53% to 77% 10-year survival, Category 3 = 5 to 6 points; 2% to 21% 10-year survival, Category 4 = more than 7 points; 0% 10-year survival (Table 1). Body mass index (BMI) was obtained during hospital admission as documented on a physical exam. Hemoglobin A1c (HbA1c) was recorded if measured within 3 months of hospitalization. Race/ethnicity was self-reported by subjects on admission (Table 1). The primary outcome assessed was intubation status. Other outcomes included hospital length of stay, intensive care unit (ICU) days, tracheostomy placement, long-term acute care facility (LTAC) discharge and inpatient mortality.

**Table 1:** Bivariate comparisons among intubated and non-intubated patients (n = 202).

	Intubated (n = 140)	Not Intubated (n = 62)	*P* value
Age in yrs (SD)	59.6(14.5)	54.3(13.8)	.02
Gender			
Male	95 (67.9)	30 (48.4)	.001
Female	45 (32.1)	32 (51.6)	
Race/Ethnicity			
Non-Hispanic Black	18 (12.9)	14 (22.6)	.30
Hispanic	79 (56.4)	28 (45.2)	
Non-Hispanic White	25 (17.9)	11 (17.7)	
Others	18 (12.9)	9 (14.5)	
Smoking History			
Yes	33 (23.6)	9 (14.5)	.80
No	107 (76.4)	48 (77.4)	
COPD			
Yes	8 (5.7)	3 (4.8)	.80
No	132 (94.3)	59 (95.2)	
Asthma			
Yes	11 (7.9)	17 (27.4)	<.001
No	129 (92.1)	45 (72.6)	
Pre-Hospital ICS			
Yes	2 (1.4)	8 (12.9)	<.001
No	138 (98.6)	54 (87.1)	
Hypertension			
Yes	84 (60.0)	33 (53.2)	.40
No	56 (40.0)	29 (46.8)	
CKDIII-ESRD			
Yes	15 (10.7)	6 (9.7)	.80
No	125 (89.3)	56 (90.3)	
Diabetes mellitus			
Yes	73 (52.1)	26 (41.9)	.20
No	67 (47.9)	36 (58.1)	
HbA1c	7.6 (1.9)	6.84 (1.5)	.03
BMI, kg/m^2^	33.6(10.6)	32.6(8.3)	.40
CCI	2.9(2.1)	2.2(2.0)	.02
CCI categories			
0	16 (11.4)	11 (17.7)	.20
1	52 (37.1)	30 (48.4)	
2	44 (31.4)	13 (21.0)	
3	17 (12.1)	5 (8.1)	
4	11 (7.9)	3 (4.8)	
SIRS	1.9(0.9)	1.7(0.8)	.10

Values are mean (SD) or number of patients (%), BMI = body mass index, CCI = charlson comorbidity index, CKDIII-ESRD = chronic kidney disease stage III to end stage renal disease, COPD = chronic obstructive pulmonary disease, HbA1c = hemoglobin A1c, ICS = inhaled corticosteroids, SD = standard deviation, SIRS = systemic inflammatory response syndrome.

### 2.3. Sample size justification

A priori power analysis was performed using the G power software (version 3.1).^[17]^ The sample size was predetermined using Cohen’s medium effect sizes (0.5 for the *t* test and 0.3 for the chi-square test), the desired power of 80%, and a 5% level of significance.^[18,19]^ The minimum sample required for the t and chi-square tests was 128 and 143, respectively. For the logistic regression, we used the formula- N ≥ 50 + 8 m, where m corresponds to the number of predictors.^[20]^ The total number of predictors was 12, according to which N = 146 was deemed appropriate. The sample size with the most outstanding value (N = 146) was considered appropriate as it satisfies the minimum requirement of all the statistical tests used in this study. The sample size used in this study was more than the minimum requirement, allowing us to derive subgroup analysis.

### 2.4. Statistical analysis

Data was first cleaned and re-coded for running analytical operations. All statistical assumptions were accessed, including normality, homogeneity of variance, and multicollinearity. Categorical variables were expressed as counts (percentages) and then compared with the chi-square analysis. Continuous variables were compared using an independent-samples *t* test and were expressed as means and standard deviations. A multivariate logistic regression model was fit to generate adjusted odds ratios for inpatient mortality and intubation. Estimates of parameters were obtained through the maximum likelihood estimation method with 95% Wald’s confidence limits for the logistic model. The final model was selected based on the Akaike Information Criterion and the Schwarz Criterion.^[21]^ For regression analyses, polytomous categorical variables were dummy coded to calculate accurate parameters. All tests were 2-sided, and a *P* value of <.05 was considered significant. The Statistical Package for Social Sciences for Windows, version 27.0 (SPSS, Chicago, IL), and Statistical Analysis System (SAS 9.4) were used to analyze the data.

## 3. Results

From March 1 to November 30, 2020, there were a total of 226 patients who presented to our tertiary teaching hospital with an emergency room diagnosis of *SARS-COVID-19*. After PCR testing for *SARS-COVID-19*, 200 and 2 patients were found to be COVID-19 PCR positive (Fig. 1). We performed a bivariate comparison of intubated and non-intubated patients. Intubated patients were older, with an average age of 59.6 years compared to their non-intubated counterparts’ average age of 54.3 (*P* = .02). Most intubated patients were males, 67.9%, while the non-intubated group consisted of a female majority, 51.6% (*P* = .001). The average BMI of the intubated group was 33.6 kg/m^2^ as opposed to 32.6 kg/m^2^ BMI among the non-intubated group, but the differences were not statistically significant (*P* = .40). Twenty-eight patients in the overall sample had a history of asthma, with 11 requiring intubation and 17 not intubated (*P* < .001). The average HbA1c in the intubated group was 7.6% compared to 6.84% in the non-intubated group (*P* = .03). Patients in the intubated group had a higher CCI when compared to those not intubated (*P* = .02). All other variables showed no statistical difference between intubated and not intubated (Table 1).

Healthcare utilization amongst intubated and not intubated patients was measured by hospital days, ICU days, and discharge to LTAC (Table 2). All intubated patients were admitted to ICU, except 1 patient who was intubated in the Emergency Department and expired. Intubated patients also required a more extended hospital and ICU stay, averaging 26.6 days and 20.0 days, respectively. In comparison, non-intubated patients were hospitalized for 10.6 days and admitted to ICU for an average of 2.5 days (*P* < .001). Amongst intubated patients, nearly 15.7% underwent tracheostomy placement, and 13.6% were discharged to LTAC (Table 2). Intubation was associated with a disposition to long-term acute care facilities (*P* = .002) and higher inpatient mortality (*P* < .001).

**Table 2:** Comparisons among intubated and non-intubated patients for healthcare utilization.

	Intubated (n = 140)	Not Intubated (n = 62)	*P* value
Hospital d (M ± SD)	26.6 (16.1)	10.6 (8.7)	<.001
ICU d (M ± SD)	20.0 (12.5)	2.5 (3.8)	<.001
Ventilator d (M ± SD)	17.4 (10.9)	1.05 (0.4)	<.001
ICU admission			
Yes	139 (99.3)	25 (40.3)	<.001
No	*1 (0.7)	37 (59.7)	
Tracheostomy			
Yes	22 (15.7)	0 (0.0)	<.001
No	118 (84.3)	62 (100)	
In-hospital mortality			
Yes	78 (55.7)	2 (3.2)	<.001
No	62 (44.3)	60 (96.8)	
LTAC			
Yes	19 (13.6)	0 (0.0)	.002
No	121 (86.4)	62 (100)	

Data are presented in mean (SD) or number of patients (%). ICU = intensive care unit, LTAC = long-term acute care, SD = standard deviation.

* Patient was intubated in the emergency department and expired before ICU admission

After logistic regression analysis, intubation was associated with male gender (odds ratio [OR], 2.815; 95% confidence interval [CI], 1.348–5.881; *P* = .006) and increasing BMI (OR, 1.053; 95% CI, 1.009–1.099; *P* = .019). Asthma was associated with lower odds for intubation (OR, 0.283; 95% CI, 0.108–0.74; *P* = .01). Other variables were not statistically significant (Table 3). In-patient mortality was associated with male gender, (OR, 2.44; 95% CI, 1.167–5.1; *P* = .018), older age (OR, 1.096; 95% CI, 1.052–1.142; *P* = <.001), and increasing BMI (OR, 1.079; 95% CI, 1.033–1.127; *P* = .001). Asthma was associated with lower in-patient mortality (OR, 0.221; 95% CI, 0.057–0.854; *P* = .029). The remaining variables were not statistically significant (Table 4).

**Table 3:** Logistic regression analysis for risk factors for intubation.

Variables	Adjusted odds ratio (95% CIs)	*P* value
Age	1.029 (0.991–1.067)	.134
Gender (vs Female)	2.815 (1.348–5.881)	.006
Hispanic vs Black	2.041 (0.751–5.543)	.162
White vs Black	1.396 (0.442–4.41)	.570
Other race vs Black	1.918 (0.549–6.697)	.307
BMI, kg/m^2^	1.053 (1.009–1.099)	.019
Smoking history	1.002 (0.428–2.344)	.997
COPD	0.534 (0.101–2.837)	.462
Asthma	0.283 (0.108–0.74)	.010
Hypertension	0.895 (0.41–1.953)	.781
Diabetes mellitus	0.988 (0.454–2.147)	.975
CKDIII- ESRD	0.572 (0.148–2.219)	.420
CCI	1.262 (0.923–1.724)	.145
SIRS	1.463 (0.993–2.156)	.054

BMI = body mass index, CCI = charlson comorbidity index, CKDIII-ESRD = chronic kidney disease stage III to end stage renal disease, COPD = chronic obstructive pulmonary disease, SIRS = systemic inflammatory response syndrome.

**Table 4:** Logistic regression analysis for risk factors for in-patient mortality.

Variables	Adjusted odds ratio (95% CIs)	*P* value
Age	1.096 (1.052–1.142)	<.001
Gender (vs Female)	2.44 (1.167–5.1)	.018
Hispanic vs Black	2.825 (0.955–8.357)	.061
White vs Black	1.73 (0.493–6.068)	.392
Other race vs Black	1.682 (0.419–6.746)	.463
BMI, kg/m^2^	1.079 (1.033–1.127)	<.001
Smoking history	0.466 (0.193–1.128)	.090
COPD	1.193 (0.228–6.247)	.834
Asthma	0.221 (0.057–0.854)	.029
Hypertension	0.75 (0.343–1.642)	.472
Diabetes mellitus	1.361 (0.619–2.992)	.444
CKDIII- ESRD	3.004 (0.783–11.528)	.109
CCI	0.896 (0.665–1.206)	.468
SIRS	1.232 (0.825–1.841)	.308

BMI = body mass index, CCI = charlson comorbidity index, CKDIII- ESRD = chronic kidney disease stage III to end stage renal disease, COPD = chronic obstructive pulmonary disease, SIRS = systemic inflammatory response syndrome.

## 4. Discussion

Asthmatics seem less prone to adverse outcomes in COVID-19 as our results showed lower odds of intubation and inpatient mortality. In contrast, more extensive studies involving COVID-NET and meta-analysis have shown that asthmatics have increased in-patient mortality. However, asthma was grouped with other chronic lung diseases.^[11,14]^ Systematic reviews and meta-analyses examining asthma alone have noted asthmatics are indeed less prone to adverse outcomes in the setting of COVID-19 infection.^[22,23]^ The center for disease control and prevention behavioral risk factor surveillance system, which monitors the prevalence of asthma in the United States, reported 230,431 asthmatic patients in the state of Nevada in 2020, approximately 9.5% of the Nevada population.^[24]^ Our study had 28 asthmatic patients admitted over 9 months, and a significant majority did not require intubation. In our bivariate comparison, more patients in the non-intubated group took ICS before hospitalization than intubated patients. However, only a small portion of the subject (n = 10) had anecdotal ICS use documented on admission and could not be used for further analysis (Table 1). Regardless 80% of patients taking pre-hospital ICS were not intubated, and this supports prior observations that anecdotal ICS use may be associated with decreased rates of intubation.^[12]^

The mechanism underlying this observation remains debatable whether anecdotal ICS or the pathophysiology of asthma itself confers some protection from COVID-19. Molecular analysis has shown that 2 airway epithelial proteins that are in part modulated by asthma (angiotensin-converting enzyme 2 [ACE-2] and the transmembrane protease serine 2 [TMPRSS2]) also facilitate SARS-CoV-2 virus entry into cells.^[25]^ Patients with asthma have been shown to express lower levels of ACE-2 in airway epithelium, which may offer some protective mechanism against COVID-19.^[26,27]^ In addition, gene expression of ACE-2 and TMPRSS2 has also been shown to be lower in sputum cells of patients with asthma on ICS, and in fact, there was a dose response.^[28]^ However, there is also data showing no differences between ACE-2 and TMPRSS2 gene expression between healthy volunteers and patients with asthma taking ICS.^[29]^ The role of anecdotal ICS use remains unclear. Still, our results support prior observations that asthmatics have fewer adverse outcomes from COVID-19, and pre-hospital ICS use may be associated with a reduced risk of intubation.

Mortality amongst intubated patients in our study was 55.7% which roughly correlates to the 53% mortality rate reported through larger observational studies in the United States.^[11]^ Intubation for COVID-19 signifies an overall poor prognosis with mortality rates exceeding more than 50%, given our results. Risk factors for inpatient mortality include older age above 50, male gender, obesity, immunosuppression, renal disease, chronic lung disease, cardiovascular disease, neurological disorders, and diabetes.^[11]^ Similarly, we found that older age, male gender, and increasing BMI were associated with adverse COVID-19 outcomes. Other comorbidities were not statistically significant; however, this does not exclude them as risk factors.

Diabetes Mellitus was not associated with adverse outcomes; however, average HbA1c was significantly higher in the intubated group than in the non-intubated. Only 150 subjects had recorded A1c levels, so this could not be used in logistic regression analysis. Regardless this supports prior observations that mortality is lower in COVID-19 patients with HbA1c < 7.0.^[30]^ The average BMI in both intubated and non-intubated groups coincided with obesity class I, which suggests that obese patients, in general, were more likely to be hospitalized for COVID-19 infection. Several mechanisms likely contribute to the role of obesity and poor glycemic control in adverse COVID-19 outcomes. Impaired immune function, chronic baseline inflammation, complement system over activation, and increased Angiotensin Converting Enzyme 2 (ACE-2) receptor expression have all been proposed as contributing factors.^[31]^ Additionally, obese patients have altered pulmonary mechanics and physiology leading to reduced lung volumes, decreased lung compliance, and respiratory muscle insufficiency that predispose this patient population to respiratory failure after pulmonary insult.^[31]^

Our results suggest that risk stratification using CCI is ineffective in predicting the likelihood of intubation or in-patient mortality for COVID-19. This is surprising given the correlation between the increasing number of comorbidities with adverse COVID-19 outcomes.^[12,13]^ Given that obesity is a significant risk factor for adverse COVID-19 outcomes^[32]^ and CCI does not incorporate this comorbidity, this raises the question that obesity may have a more substantial role in predicting prognosis in COVID-19 than other common comorbid conditions. In addition, the results support a single-center study from Brazil showing that systemic inflammatory response syndrome was a poor prognosticator for predicting inpatient mortality.^[33]^ Several other COVID-19-specific risk stratification tools have been developed and should be considered for patients with COVID-19 infection.^[34–37]^

This study is limited by its retrospective design and small sample size, which weakens the statistical power. Given that this is a single-center study, the results may represent a local medical practice that may not be generalizable. Treatment courses varied as new COVID-19 treatments became available via emergency Federal Drug Administration authorizations, but no patients included in the study received COVID-19 vaccinations. Chronic obstructive pulmonary disease and asthma did not specify illness severity, and there was insufficient anecdotal ICS use documented for further analysis. This study was not associated with any funding sources.

In conclusion, older age, male gender, and increasing BMI were associated with worse COVID-19 outcomes. These findings alert healthcare providers to high-risk populations that would benefit from targeted vaccination efforts. CCI score was not associated with intubation or inpatient mortality. This risk assessment tool does not consider obesity which remains a significant risk factor for adverse COVID-19 outcomes. Asthmatic patients were less likely to undergo intubation and had lower in-patient mortality if hospitalized for COVID-19, and most patients taking pre-hospital ICS were not intubated. Further hypothesis-driven studies using a larger sample size must confirm these findings.

## Author contributions

**Conceptualization:** Andrew Manfra, Kyaw Min Tun, Mutsumi John Kioka.

**Data curation:** Andrew Manfra, Claire Chen.

**Formal analysis:** Kavita Batra.

**Investigation:** Kyaw Min Tun, Mutsumi John Kioka.

**Methodology:** Andrew Manfra, Kavita Batra.

**Project administration:** Andrew Manfra, Mutsumi John Kioka.

**Software:** Kavita Batra.

**Supervision:** Mutsumi John Kioka.

**Writing – original draft:** Andrew Manfra, Claire Chen.

**Writing – review & editing:** Andrew Manfra, Kyaw Min Tun, Mutsumi John Kioka.
